# Retraction Note: Growth inhibition and apoptosis induced by 6-fluoro-3-formylchromone in hepatocellular carcinoma

**DOI:** 10.1186/s12876-015-0265-7

**Published:** 2015-03-26

**Authors:** Yijie Zhang, Kailian Zheng, Hongli Yan, Gang Jin, Chenghao Shao, Xuyu Zhou, Yingqi Zhou, Tianlin He

**Affiliations:** Department of General Surgery, Changhai Hospital, No.168 Changhai Road, Yangpu District, 200433 Shanghai, China

## Retraction

The Publisher and Editor regretfully retract this article [1] because the peer-review process was inappropriately influenced and compromised. As a result, the scientific integrity of the article cannot be guaranteed. A systematic and detailed investigation suggests that a third party was involved in supplying fabricated details of potential peer reviewers for a large number of manuscripts submitted to different journals. In accordance with recommendations from COPE we have retracted all affected published articles, including this one. It was not possible to determine beyond doubt that the authors of this particular article were aware of any third party attempts to manipulate peer review of their manuscript.

## References

[1] Zhang Y, Zheng K, Yan H, Jin G, Shao C, Zhou X (2014). Growth inhibition and apoptosis induced by 6-fluoro-3-formylchromone in hepatocellular carcinoma. BMC Gastroenterol.

